# Differential expression and regional distribution of aquaporins in amnion of normal and gestational diabetic pregnancies

**DOI:** 10.14814/phy2.12320

**Published:** 2015-03-05

**Authors:** Amy D Bednar, Michael K Beardall, Robert A Brace, Cecilia Y Cheung

**Affiliations:** 1 Division of Maternal Fetal Medicine, Department of Obstetrics & Gynecology, Oregon Health and Science UniversityPortland, Oregon; 2 Center for Developmental Health, Oregon Health and Science UniversityPortland, Oregon

**Keywords:** Amniotic fluid volume, aquaporins, gestational diabetes, human term amnion

## Abstract

The region of the amnion overlying the placenta plays an active role in fluid exchange between amniotic fluid and fetal blood perfusing the surface of the placenta, whereas little transfer occurs across the reflected amnion that contacts the membranous chorion. Because aquaporins (AQPs) facilitate rapid movement of water across cells, we hypothesized that AQP gene expression in placental amnion is higher than in reflected amnion. Furthermore, because gestational diabetes mellitus (GDM) is often associated with polyhydramnios, we hypothesized that amnion AQP gene expression is reduced when amniotic fluid volume is elevated. Human placental and reflected amnion were obtained at cesarean delivery and subjected to relative quantitation of AQP mRNA by real-time RT-qPCR and proteins by western immunoblot. Amnion mRNA levels of five AQPs differed by up to 400-fold (*P* < 0.001), with AQP1 and AQP3 most abundant, AQP8 least and AQP9 and AQP11 intermediately expressed. Aquaporin proteins showed a similar profile. Aquaporin mRNA abundance was higher (*P* < 0.001) in placental than reflected amnion, whereas protein levels were lower (*P* < 0.01). In GDM pregnancies, neither AQP mRNA nor protein levels were different from normal. There was no correlation between AQP mRNA or protein levels with the amniotic fluid index in normal or GDM subjects. We conclude that there is a strong differential expression profile among individual AQPs and between regions of the amnion. These findings suggest differences in contribution of individual AQPs to water transport in the two regions of the amnion. Furthermore, AQP expression in the amnion is not altered in patients with GDM.

## Introduction

Amniotic fluid volume (AFV) is regulated primarily by modulating the rate of transfer of amniotic water and solutes across the amnion into fetal circulation (Gilbert and Brace 1989; Anderson et al. 2013; Brace et al. 2014). This transport process is referred to as intramembranous absorption (IMA) and has two volume components (Brace et al. 2004): (1) a unidirectional vesicular transcytotic transport of amniotic water with dissolved solutes outward across the amnion cell; and (2) a passive diffusion of water down osmotic gradients from amniotic fluid into fetal blood presumably through aquaporin (AQP) channels. AQPs are integral membrane proteins that form channels to facilitate rapid movement of water and a few small molecules across cell membranes (Agre et al. 1993; Hachez and Chaumont 2010). Of the 13 known mammalian AQP genes, five (AQP1, AQP3, AQP8, AQP9, and AQP11) are known to express in the human amnion (Wang et al. 2001, 2004; Mann et al. 2002; Mobasheri et al. 2005; Damiano 2011; Prat et al. 2012). Since early observations of AQPs presence in the amnion, it has been speculated (Liu and Wintour 2005; Beall et al. 2007a) that the AQPs may play a significant role in regulating amniotic water transport across the amnion and thus may be an important regulator of AFV. However, the role of AQPs in regulating AFV is poorly understood. To address this gap in understanding, the relative expression of each AQP in the amnion needs to be explored. Thus, in this study, we hypothesized that the level of expression of the 5 AQPs in the amnion differs, with the highly selective and most efficient classic AQP1 water channel being most expressed relative to the other AQPs.

In humans, IMA occurs across that portion of the amnion that overlays the placenta (placental amnion) where amniotic fluid is transported from the amniotic cavity into fetal blood vessels on the surface of the placenta (Seeds 1980), whereas minimal transfer occurs across the amnion overlying the membranous chorion (reflected amnion) (Seeds 1980; Benirschke and Kaufmann 2000). Because of the regional differences in fluid transport behavior together with the reported regional differences in gene expression profile within the amnion (Han et al. 2008), we speculate the AQP gene expression in the two regions of the amnion would differ. However, in previous studies that explored AQP expression in human amnion, either the regional source of the amnion studied was not specified (Wang et al. 2001, 2004) or comparisons of the relative expression levels between the two regions of the amnion were not made (Mann et al. 2002; Prat et al. 2012). Furthermore, these studies focused on AQP mRNA levels but not the proteins that mediate biological functions. Thus, we undertook this study to test the hypothesis that the placental amnion would express higher levels of AQP proteins than reflected amnion, and that AQP mRNA levels would be positively correlated with protein levels.

Presently, it remains unclear whether AQPs play a role in the etiology of AFV abnormalities. In patients with abnormally high AFVs (polyhydramnios), AQP1, AQP8, and AQP9 mRNA levels in the amnion were reported to be elevated (Mann et al. 2006; Zhu et al. 2010), whereas patients with oligohydramnios (AFVs below normal) have reduced AQP1, AQP3, AQP8, and AQP9 mRNA expression (Zhu et al. 2009; Jiang et al. 2012). Based on these observations, it has been postulated that changes in AQP expression represent adaptive responses to rather than causes of AFV aberrations (Beall et al. 2007b; Zheng et al. 2014). However, the majority of these studies analyzed AQP gene expression in reflected amnion rather than placental amnion where IMA occurs (Seeds 1980). Furthermore, AQP protein levels were not determined. One approach to address the issue of cause versus consequence is to examine AQP levels in the presence of disease but without the aberration in AFV. Gestational diabetes mellitus (GDM) is a frequent pregnancy complication associated with increased incidence of polyhydramnios (Sohaey et al. 1994; McMahon et al. 1998), although the majority of GDM patients are clinically managed to control maternal circulating glucose levels and have normal AFVs. In those subjects that develop polyhydramnios, fetal hyperglycemia-induced polyuria has been suggested as the cause of the increased AFV (Dashe et al. 2000; Santolaya-Forgas et al. 2006). However, reduced trans-amnion water transport due to reduced AQP gene expression in GDM could also be involved. To explore this possibility, we tested the hypothesis that AQP expression in placental amnion of GDM patients is negatively correlated with the amniotic fluid index (AFI), a measure of AFV.

## Materials and Methods

### Study subjects and inclusion criteria

The study design was approved by the Institution Review Board of Oregon Health and Science University (OHSU) and written Informed Consent and HIPPA Research Authorization was obtained from all subjects that participated in the study. Twenty subjects with normal pregnancies and 13 subjects with GDM were recruited on Labor and Delivery at OHSU. Diagnosis of GDM was based on a 2-h oral glucose tolerance test (OGTT) at 24- to 28-week gestation. Of the 13 GDM subjects, seven were diet-controlled (A1DM) and six were on oral medication (A2DM). Inclusion criteria were as follows: singleton pregnancies at term (37–40 weeks), an AFI of 8–25 cm (Phelan et al. 1987), absence of active labor or chorioamnionitis, and delivery via cesarean section. An AFI was obtained on the day of the cesarean section (*n* = 29) except for four patients whose AFI was obtained within 1 month of delivery.

### Amnion tissue collection

Fetal membranes were collected immediately after cesarean delivery. Placental amnion was separated from the underlying placenta and samples (approximately 2 × 4 cm each) were obtained centrally away from the umbilical cord insertion and the edge of the placenta. The reflected amnion was separated from the chorion and samples obtained distal to the placenta and incision site. For each subject, seven representative tissue samples were collected from each region of the amnion. All tissues were rinsed in DMEM/F12 (Invitrogen, Life Technologies, Grand Island, NY) to remove blood and other contamination. Amnion samples were either placed in RNA*later* solution (Ambion, Life Technologies, Carlsbad, CA) or snap frozen in liquid nitrogen for storage at −80°C until further analysis.

### Reverse transcription and real-time PCR for aquaporins

Amnion tissue samples stored in RNA*later* were homogenized in a bead-mill TissueLyser (Retsch GMBH & Co., Haan, Germany) and RNA extracted using an RNeasy Kit (Qiagen, Inc., Valencia, CA). Single-strand cDNA synthesis was carried out using MultiScribe reverse transcriptase (50 U/*μ*L) and random primers with RNase inhibitor (Applied Biosystems, Life Technologies, Foster City, CA). Sample cDNA (0.6 to 1.5 *μ*L at 50 ng/*μ*L) was amplified using TaqMan master mix with predesigned Gene Expression Assays for human AQP1, AQP3, AQP8, AQP9, or AQP11 (Applied Biosystems). The internal reference used was 18S ribosomal RNA. Validation studies were performed prior to analysis of experimental samples to confirm that 18S expression level was not different between placental and reflected amnion. A ViiA7 real-time PCR system (Applied Biosystems) was used with a temperature profile of an initial two-step hold at 50°C for 2 min and 95°C for 10 min, followed by 40 cycles of 15 sec at 95°C and 1 min at 60°C. Aquaporin gene expression was referenced to 18S ribosomal RNA and quantified by the Comparative *C*_T_ method. The relative quantity of target mRNA was calculated using the formula 2^−ΔΔ*C*^_T_ and fold change was determined using placental AQP8 as the calibrator. A calibrator was used in order to allow comparison of normalized mRNA levels among the five AQPs. Placental AQP8 was chosen because our data showed that its expression level was the lowest among the five AQPs in the two regions of the amnion. To compare relative expression levels among the AQPs, the calculated fold change values (with placental AQP8 as calibrator) were used for statistical analysis. The validity of this methodology was based on (1) the 100 ± 10% amplification efficiency for each of the five human AQP Taqman assays and (2) comparable sensitivity (as defined by the *C*_T_ value of the minimum detectable copy number for each AQP template being significantly less than (*P* < 0.05) the no-template control) among the five AQP assays at 40 cycles of amplification (Applied Biosystems).

### Western immunoblot for aquaporin proteins

Frozen amnion tissues were homogenized in the bead-mill TissueLyser and membrane proteins were extracted using a Native Membrane Protein Extraction Kit (Calbiochem, EMD Millipore, Billerica, MA). Membrane proteins (30 *μ*g/sample) were subjected to SDS-PAGE (sodium dodecyl sulfate-polyacrylamide gel electrophoresis) in pre-cast 12% polyacrylamide gels (Bio-Rad Laboratories, Hercules, CA) and electro-transferred onto PVDF membranes (0.45 *μ*m, EMD Millipore). The PVDF membranes were blocked with 5% (w/v) nonfat dry milk in TBS-Tween 20 (pH 7.6) and incubated with the primary antibody at 4°C overnight. For the detection of AQP proteins, specific anti-human antibodies (Santa Cruz Biotechnology, Inc. Santa Cruz, CA) for AQP1, AQP3, AQP8, AQP9, and AQP11 were used at dilutions indicated in Table1. This was followed by incubation with horseradish peroxidase-conjugated secondary antibodies (Table1) for 45 min at room temperature. Protein expression was detected using enhanced chemiluminescent substrate (Pierce® ECL2 Western Blotting Substrate, Thermo Scientific) and exposure to autoradiography using BlueUltra autoradiography film (GeneMate, BioExpress, Kaysville, UT). Beta-actin was used as the endogenous control. The PVDF membranes were stripped by incubating in antibody stripping buffer (ReBlot Plus, EMD Millipore, Billerica, MA) for 20 min at room temperature to dissociate the AQP antibody from the membrane and reprobed with an anti-human *β*-actin antibody (Table1) and similarly treated. The autoradiograms were scanned, images captured, and intensity of the signal for the specific protein was determined by densitometry using Image Studio Lite software (Version 3.1, Li-Cor Bioscience, Lincoln, NE).

**Table 1:** Human-specific antibodies for immunoblotting of placental and reflected Amnion – Santa Cruz Biotechnology, Inc

Target Protein	Primary antibody product no.	Isotype	Dilution	Secondary antibody product no.	Isotype	Dilution
AQP1	sc-9878 (L-19)	Goat polyclonal IgG	1:500	sc-2304	Donkey anti-goat HRP polyclonal IgG	1:40,000
AQP3	sc-9885 (C-18)	Goat polyclonal IgG	1:500	sc-2304	Donkey anti-goat HRP polyclonal IgG	1:40,000
AQP8	sc-28624 (H-85)	Rabbit Polyclonal IgG	1:20,000	sc-2054	Goat anti-rabbit HRP polyclonal IgG	1:40,000
AQP9	sc-74409 (G-3)	Mouse monoclonal IgG2a	1:500	sc-2970	Goat anti-mouse HRP monoclonal IgG2a	1:40,000
AQP11	sc-138132 (N-14)	Goat polyclonal IgG	1:500	sc-2304	Donkey anti-goat HRP polyclonal IgG	1:40,000
*β*-actin	sc-69879 (AC-15)	Mouse Monoclonal IgG1	1:1.25 × 10^6^	sc-2969	Goat anti-mouse HRP monoclonal IgG1	1:40,000

For quantitative comparison of protein levels among individual AQPs and between amnion regions, a standard curve was incorporated in each SDS-PAGE assay. The standard curve was generated using a pooled protein mix of placental and reflected amnion in the amounts of 1, 3, 10, and 30 *μ*g. The same pooled protein mixture was used for all the standard curves in this study. The densitometric values of the standards were determined by nonlinear regression to generate a standard curve. Sample AQP and *β*-actin values were calculated from the standard curves and the relative protein quantity of individual AQP was referenced to the respective quantity of *β*-actin before comparative analysis.

### Statistical analysis

Data are presented as mean ± SEM. One-factor analysis of variance (ANOVA) was used to compare mRNA or protein levels among the AQPs within each region of the amnion. Comparison of mRNA or protein quantities between placental and reflected amnion, and between normal and GDM subjects in each amnion region, were performed by two-factor ANOVA followed by Fisher's least significant difference test. Bivariate regression was used to determine relationships between mRNA and protein levels. A one-factor analysis of covariance (ANCOVA) was used for the comparison of mRNA versus protein relationships between the two regions of the amnion. Bivariate and multivariate regression was applied for the analysis of relationships between the AQPs and AFI. Base 10 log transformation was performed when necessary to normalize variances prior to analysis. *T*-tests and chi-square tests were used to compare patient demographics. *P* ≤ 0.05 was accepted as statistically significant.

## Results

### Demographics of study subjects

The demographics of the 20 subjects with normal pregnancies and 13 GDM subjects with normal AFI are shown in Table2. In the GDM subjects, all but two were well controlled while all had AFI within the normal range. There were no significant differences in patient demographics between normal and GDM subjects and between A1DM and A2DM subjects.

**Table 2:** Demographics of study subjects at cesarean section

	Normal (*n* = 20)	Gestational diabetes mellitus (*n* = 13)	*P* value*
Gestational age (weeks)	39.0 ± 0.2	39.0 ± 0.1	1.0
Maternal age (years)	30.5 ± 1.6	32.5 ± 1.5	0.40
BMI (kg/m^2^)	32.4 ± 1.4	36.0 ± 2.3	0.16
AFI (cm)	11.9 ± 0.6 (*N* = 17)	14.0 ± 1.3	0.12
Birth weight (grams)	3461 ± 107	3532 ± 143	0.67
Neonatal gender	Male (*n* = 11) Female (*n* = 9)	Male (*n* = 2) Female (*n* = 9)	0.07

BMI, body mass index; AFI, amniotic fluid index.

The gestational diabetes mellitus group consisted of seven A1DM subjects and six A2DM subjects. The two groups were combined for analysis because there was no difference in any of the demographic parameters.

* *P* values determined by unpaired *t*-test or chi-square test.

### Regional differences in aquaporin mRNA expression in normal pregnancies

**Figure 1:**
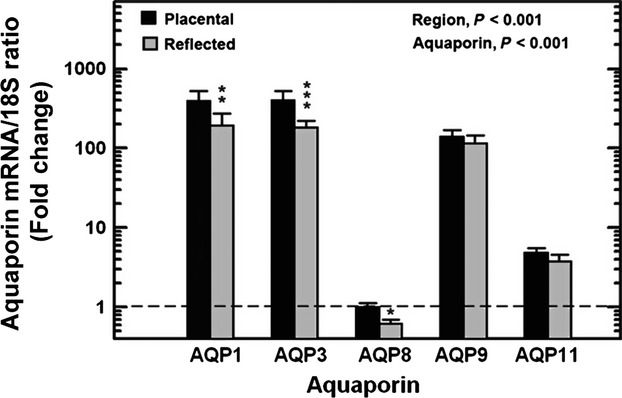
Regional expression pattern of five aquaporin mRNAs in human amnion. Aquaporin mRNA levels in placental and reflected amnion were normalized to 18S internal reference and expressed as fold change (mean ± SEM, *n* = 20) from the respective placental AQP8 mRNA level (calibrator, marked by horizontal dashed line). The data were analyzed by a two-factor ANOVA. The difference in mRNA levels among the five aquaporins within each region of the amnion was significant at *P* < 0.001. The overall difference in aquaporin mRNA levels between placental and reflected amnion was significant at *P* < 0.001. Post hoc testing showed differences between placental and reflected amnion for AQP1, AQP3 and APQ8. ***P < 0.001; **P < 0.01; *P < 0.05.

In placental amnion of normal subjects, there were large differences among individual AQP mRNA levels (Fig.1). AQP1 and AQP3 levels were 400 fold, whereas AQP9 and AQP11 averaged 140 and 5 fold that of AQP8, respectively (*P* < 0.001). In reflected amnion (Fig.1), the mRNA levels were 195, 182, 0.62, 116, and four fold that of placental AQP8 for AQP1, AQP3, AQP8, AQP9, and AQP11, respectively (*P* < 0.001). Although the expression profile appeared qualitatively similar, AQP mRNA levels in placental amnion were significantly higher than those in reflected amnion by an overall mean average of 1.6 fold (*P* < 0.001, Fig.1). With post hoc testing, AQP1, AQP3, and AQP8 mRNA levels were significantly different at 2.0, 2.2, and 1.6 fold, respectively, in placental compared to reflected amnion.

### Aquaporin protein levels and regional differences in normal pregnant subjects

**Figure 2:**
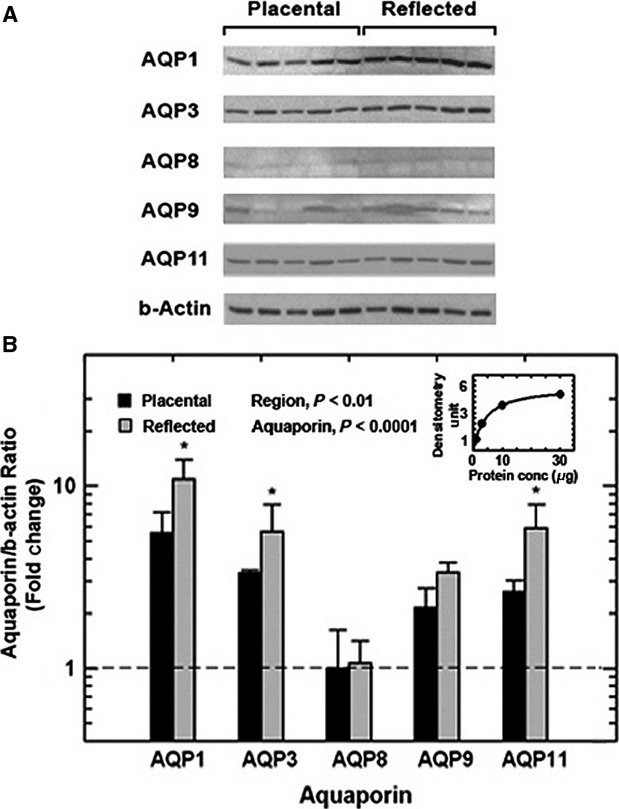
Aquaporin protein expression in normal human placental and reflected amnion. (A) Photomicrographs of immunoblots for AQP1, AQP3, AQP8, AQP9, and AQP11 in placental and reflected amnion membrane proteins. A representative *β*-actin image is shown. For each aquaporin, the protein band shown was detected at a molecular weight specific for the antibody used (AQP1, 35 kD; AQP3, 36 kD; AQP8, 34 kD; AQP9, 33 kD; and AQP11, 36 kD; *β*-actin, 43 kD. Published data by Santa Cruz Biotechnology, Inc.). (B) Aquaporin protein quantities relative to *β*-actin internal reference was expressed as fold change (mean ± SEM, *n* = 5) from the mean placental AQP8 level as the calibrator (marked by horizontal dashed line). For comparison of protein levels among individual aquaporins, densitometric values of the aquaporin and the respective *β*-actin were calculated from the respective protein standard curves (a representative standard curve shown in the insert). The data were analyzed by a two-factor ANOVA. Statistical significances were obtained between amnion regions (*P* < 0.01) and among the five aquaporins within each region (*P* < 0.0001). *P < 0.05, by post hoc testing between placental and reflected amnion for AQP1, AQP3 and AQP11.

In placental amnion, there were significant differences among AQP protein levels (*P* < 0.0001, Fig.2B), with relative quantities of AQP1 and AQP3 highest, while AQP8 the lowest at levels approaching the limit of detectability (Fig.2A, B). This protein expression profile was comparable to that of AQP mRNA levels. In the reflected amnion, AQP proteins displayed a similar pattern with large differences among individual proteins (*P* < 0.0001, Fig.2A, B). Comparisons between regions showed that AQP protein levels were significantly higher in reflected than placental amnion (*P* < 0.01, Fig.2B). AQP1, AQP3, and AQP11 in the reflected amnion were 2.0, 2.2, and 2.5 fold, respectively, of that in placental amnion.

### Relationship between aquaporin mRNA and protein levels in normal pregnant subjects

**Figure 3:**
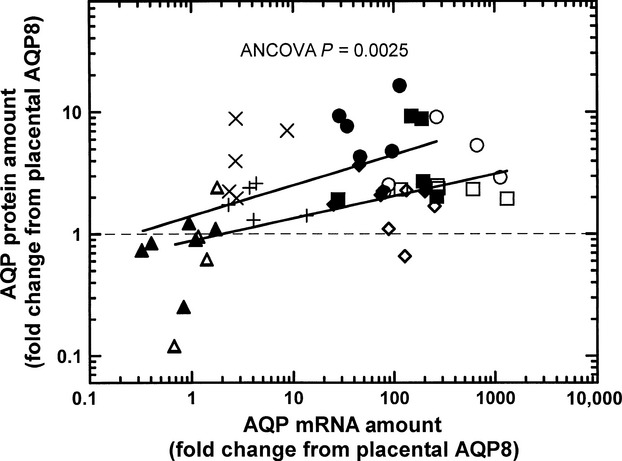
Comparison of aquaporin protein and mRNA relationship between placental and reflected amnion. Lower regression line with open symbols denotes relationship in placental amnion and upper regression line with filled symbols for reflected amnion. Circles, AQP1; squares, AQP3; triangles, AQP8, diamonds, AQP9; crosses and pluses, AQP11. The analysis of covariance (ANCOVA) indicated that the aquaporin protein versus mRNA relationship was significantly different between placental and reflected amnion (*P* < 0.01).

There was a significant but weak log–log relationship between AQP protein (Y) and mRNA (X) levels in placental amnion: log(Y) = −0.053 + 0.186 log(X), *r* = 0.57, *P* = 0.0028. In the reflected amnion, a similar log–log relationship was obtained: log(Y) = 0.151 + 0.255 log(X), *r* = 0.57, *P* = 0.0032. A comparison of these two relationships showed that, per unit mRNA, the amount of protein expressed was greater in reflected than placental amnion (*P* < 0.01, ANCOVA, Fig.3).

### Aquaporin expression and amniotic fluid volume relationship

In normal pregnant subjects, the AFI varied from 8.6 to 15.6 cm. Neither mRNA nor protein levels for the five AQPs in either placental or reflected amnion were significantly correlated with AFI. Furthermore, multivariate regression analysis of all five AQPs simultaneously did not yield statistically significant relationships between the level of AQP expression and the AFI.

### Aquaporin gene expression in gestational diabetic subjects

**Figure 4:**
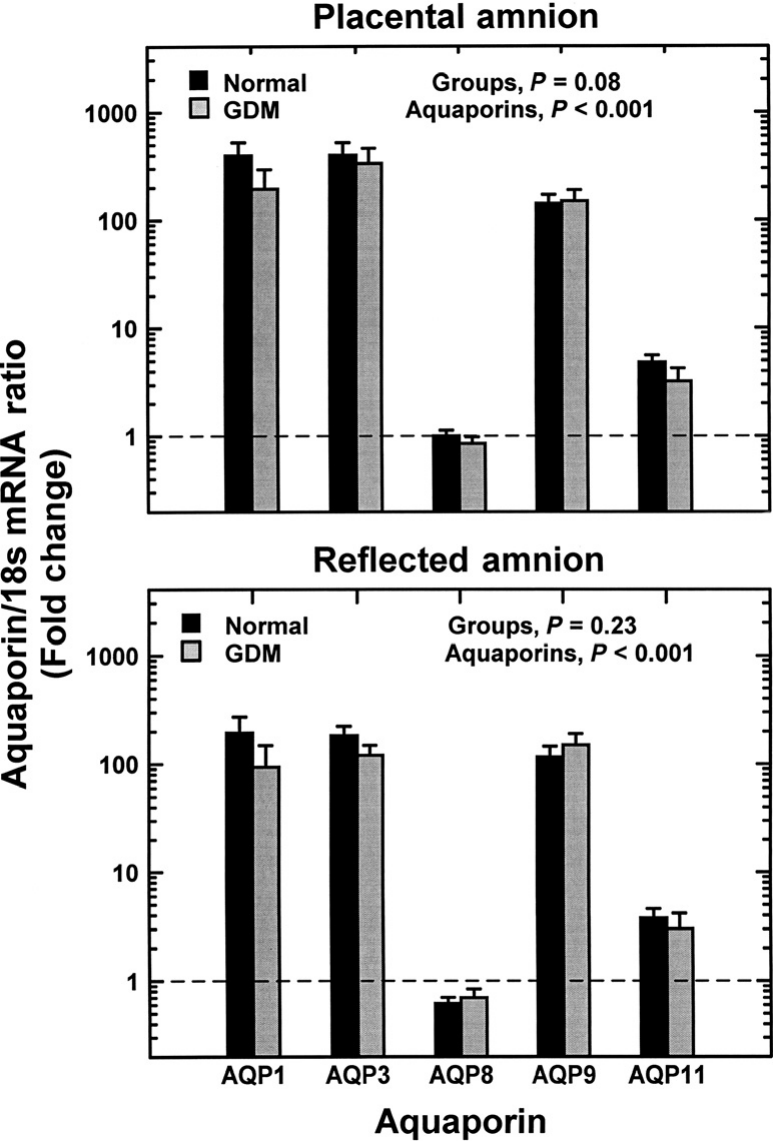
Aquaporin gene expression profile in placental (upper panel) and reflected (lower panel) amnion of normal (*n* = 20) and gestational diabetic subjects (*n* = 13). Aquaporin mRNA levels in placental and reflected amnion were normalized to 18S internal reference and expressed as fold change (mean ± SEM) from placental AQP8 mRNA of normal subjects (calibrator, marked by horizontal dashed line). The data were analyzed by a two-factor ANOVA. Within each region, individual mRNA levels differed (*P* < 0.001). Individual aquaporins were not different between normal and gestational diabetic subjects in either placental or reflected amnion.

In the GDM subjects with normal AFI, those under diet control and those on oral medication had similar AQP mRNA levels for both regions of the amnion, thus data for the two GDM groups were combined for analysis. In placental amnion of GDM subjects, the mRNA expression profile of the five AQPs was similar to that in placental amnion of normal subjects (*P* = 0.08), with AQP1 and AQP3 highest while AQP8 was lowest. There were no significant differences in individual AQP mRNA levels between normal and GDM subjects (Fig.4, upper panel). A similar profile and lack of significant differences (*P* = 0.23) in AQP mRNA levels were found in the reflected amnion as compared to normal subjects (Fig.4, lower panel). Comparison between amnion regions of GDM subjects showed significantly higher AQP mRNA levels in placental than reflected amnion (*P* = 0.03), similar to the regional differences in normal subjects. In placental and reflected amnion of GDM subjects, AQP1 and AQP3 protein levels were high, while AQP8 levels were least with profiles of the five AQPs similar to those in normal subjects (data not shown).

There were no significant correlations between AFI and AQP mRNA or protein levels in either region of the amnion in GDM subjects. For the normal and GDM patients combined, AFI varied from 8.6 to 23.0 cm and remained uncorrelated with AQP mRNA and protein levels. This lack of relationship between AFI and AQP mRNA or protein levels was not altered by exclusion of data from the four individuals in which AFI was measured prior to the day of delivery.

## Discussion

An important finding in this study is that a highly significant differential expression profile exits among the five AQPs in the two regions of the human amnion, and this occurs in normal as well as GDM subjects. This unique observation implies that each AQP may have a different function with specific roles in transport. With AQP1 and AQP3 mRNA and protein levels up to 400 fold that of AQP8, these two AQPs likely play a dominant role in passive water movement across the amnion. Although previous studies have reported the gene expression of AQP1, AQP3, AQP8, AQP9, and AQP11 in human amnion, those findings were obtained from separate studies of one or two AQPs (Wang et al. 2001, 2004; Mann et al. 2002; Mobasheri et al. 2005), or reporting mRNA levels without simultaneous protein determinations (Wang et al. 2001, 2004; Mann et al. 2002; Damiano 2011). Furthermore, there were no comparisons of expression level of mRNA or protein among individual AQPs or between amnion regions were made (Prat et al. 2012). Our study differed in that we designed the study with the purpose of comparing both the mRNA and protein expression levels among the five AQPs and analyzing the differences in expression profile between the two regions of the amnion. In addition, we explored whether the AQP expression profile would be altered by the maternal condition of GDM. Our findings support our hypothesis that AQP1 and AQP3 are highly expressed in the human amnion. Further studies are needed to compare the relative water conductance of individual AQP water channels in the amnion.

The current understanding of amniotic water transport suggests that the transport function of placental and reflected amnion differs with rapid water movement across the placental amnion into the underlying fetal circulation, whereas minimal water movement occurs across the reflected amnion (Seeds 1980; Brace et al. 2014). Our finding that AQP mRNA levels are higher in placental than reflected amnion supports the concept of regional differences in transport function. Furthermore, the difference in mRNA levels is consistent not only with the report of regional heterogeneity in gene expression profile in human amnion (Han et al. 2008) but also with our hypothesis that AQP mRNA levels are higher in the placental than reflected amnion because of higher water transport rates.

However, an unexpected observation is that AQP protein levels are lower in placental than reflected amnion even though AQP mRNA levels are higher. This difference was detected by both the ANOVA comparison of relative protein quantities as well as with the ANCOVA comparison of protein levels relative to mRNA levels. The reason for the lower placental AQP protein levels remains unknown. It may be due to inherent differences in the translation of AQP mRNA into protein within the two regions of the amnion. Translation of genes from mRNA into functional protein is a complex process involving multiple levels of translational regulation as well as posttranslational modifications (Arcondéguy et al. 2013). An additional possibility, although speculative, is that, due to the high rate of water transfer in placental amnion, the turnover and/or degradation of AQP proteins could be increased thus reducing the steady-state levels of AQP proteins. This is analogous to the arginine vasopressin induced increase in translocation of AQP2 out of the apical membrane of renal tubules resulting in elevated urinary excretion of AQP2 (Elliot et al. 1996).

Previous studies have reported discordance between mRNA and protein expressions (Prat et al. 2012). In this study, we found, as hypothesized, a modest positive correlation (*r*^2^ × 100% = 32%) between AQP protein and mRNA levels. This suggests that two-third (68%) of the variations in AQP protein quantities do not correspond to mRNA levels. It should be noted that our analytical methods for relative quantitation of AQP mRNA utilized the comparative *C*_T_ method based on 100% PCR amplification efficiency while protein levels were determined semiquantitatively using a protein standard curve. It is possible that correspondence would have been higher if the protein levels were more quantitatively determined. Irrespective, the modest correspondence between transcript and protein emphasizes the need for determination of protein levels in addition to mRNA in AQP gene function studies. In previous studies that reported changes in AQP mRNA expression in amnion of patients with abnormal AFV (Mann et al. 2006; Zhu et al. 2009, 2010; Jiang et al. 2012), it would be of particular interest to ascertain if AQP protein levels were also altered. This perspective is highlighted by our finding that the quantity of individual AQP protein per unit mRNA was significantly lower in placental amnion where IMA occurs than in the reflected amnion.

Previous studies have reported increases in AQP mRNA levels with polyhydramnios and decreases with oligohydramnios in the amnion (Mann et al. 2006; Zhu et al. 2009, 2010; Jiang et al. 2012). The pregnancy complication of GDM is often associated with polyhydramnios (Cardwell 1987; Golan et al. 1994). However, for the majority of GDM patients who have a normal AFV, it was unknown whether amnion AQP gene expression would change and whether AQP levels would correlate with AFV. Our findings indicated that AQP mRNA and protein levels in both placental and reflected amnion of GDM patients were not different from those in normal pregnant subjects, and that there was no correlation between AQP expression level and AFI when the AFI is in the range accepted as clinically normal (Phelan et al. 1987). This observation suggests that maternal hyperglycemia alone may not have significant effects on AQP expression in the amnion.

Although the lack of correlation between AFI and AQP mRNA or protein levels in this study suggests that AQPs may not play a major role in regulating AFV, this suggestion should be interpreted cautiously. First, the AFI, although routinely used clinically to estimate AFV, is only a semiquantitative estimate. Second, neither oligohydramnios nor polyhydramnios was present in our patient population. Other investigators have reported both increases and decreases in AQP mRNA levels when AFV is outside the normal range (Mann et al. 2006; Zhu et al. 2009, 2010; Jiang et al. 2012). Furthermore, our earlier study showed that the addition of water to the ovine amniotic compartment induced rapid IMA of water (Gilbert and Brace 1989). Such observations suggest that AQP water channels in the amnion do appear to contribute to water transport across the amnion. However, this contribution may be small compared to net IMA rate in view of the fact that the water diffusion component of amniotic fluid transport constitutes approximately 15–20% of overall intramembranous transport (Brace et al. 2014).

In summary, our study demonstrates a highly significant differential expression profile among individual AQP mRNAs and AQP proteins as well as regional differences in expression pattern for the five AQPs in normal human term amnion. The contrasting results of higher AQP mRNA levels and lower AQP protein levels in the placental compared to reflected amnion points to the need for investigating the transport function of individual AQPs in both regions of the amnion. Finally, the finding that amnion AQP mRNA and protein levels in GDM patients were not different from normal subjects, together with the absence of correlation between AFI and AQP expression levels, is consistent with the concept that AQP expression in the amnion may be responsive to changes in AFV status rather than a cause of AFV abnormalities.
